# Robot-assisted transhiatal lower esophagectomy and proximal gastrectomy for Siewert type II advanced esophagogastric junction cancer with situs inversus totalis: a case report

**DOI:** 10.1186/s40792-022-01393-x

**Published:** 2022-03-14

**Authors:** Kaoru Katano, Noriyuki Inaki, Takahisa Yamaguchi, Hiroto Saito, Mari Shimada, Shiro Terai, Koichi Okamoto, Hideki Moriyama, Jun Kinoshita, Keishi Nakamura, Itasu Ninomiya

**Affiliations:** Department of Gastrointestinal Surgery/Breast Surgery, Kanazawa University Graduate School of Medical Sciences, 13-1 Takara-machi, Kanazawa, Ishikawa 920-8641 Japan

**Keywords:** Situs inversus totalis, Robotic surgery, Esophagogastric junction cancer, Siewert type II cancer, And Transhiatal esophagectomy

## Abstract

**Background:**

Situs inversus totalis (SIT) is a rare congenital abnormality in which the thoracic and abdominal organs are reversed or mirrored from their usual positions. We herein report the first case of robot-assisted transhiatal lower esophagectomy and proximal gastrectomy with esophagogastrostomy for treatment of Siewert type II advanced esophagogastric junction (EGJ) cancer with SIT.

**Case presentation:**

A 62-year-old man with SIT and intestinal malrotation was diagnosed with T3N0M0 Stage IIA EGJ cancer. Three-dimensional reconstruction of a computed tomography angiogram showed that the common hepatic artery was absent, the proper hepatic artery was derived from the superior mesenteric artery through the gastroduodenal artery, and an accessary left hepatic artery arose from the left gastric artery. The patient underwent robot-assisted transhiatal lower esophagectomy and proximal gastrectomy with D2 lymph node dissection, including lower mediastinal lymphadenectomy. Intraoperative examination revealed minor vascular abnormalities, including three branches of the left gastric artery and two left gastric veins, that had not been recognized preoperatively. The surgery was performed safely, and the patient had an uneventful postoperative course.

**Conclusions:**

Robotic-assisted surgery is efficient even for complex conditions, such as Siewert type II advanced EGJ cancer with SIT.

## Background

Situs inversus totalis (SIT) is a congenital abnormality in which the thoracic and abdominal organs are reversed or mirrored from their usual positions. It is a relatively rare condition that occurs in 1 of every 5000 to 20,000 people [1, 2]. In patients with SIT, surgery for gastric cancer can be technically difficult and confusing because of anatomical anomalies, including organ location abnormalities [3]. Robot-assisted gastrectomy has recently become widespread, and evidence has shown its feasibility and superiority [4–6]. It has also been applied to difficult procedures such as esophageal cancer and esophagogastric junction (EGJ) cancer. We herein report a case of robot-assisted transhiatal lower esophagectomy and proximal gastrectomy for Siewert type II advanced EGJ cancer with SIT.

## Case presentation

**Fig. 1:**
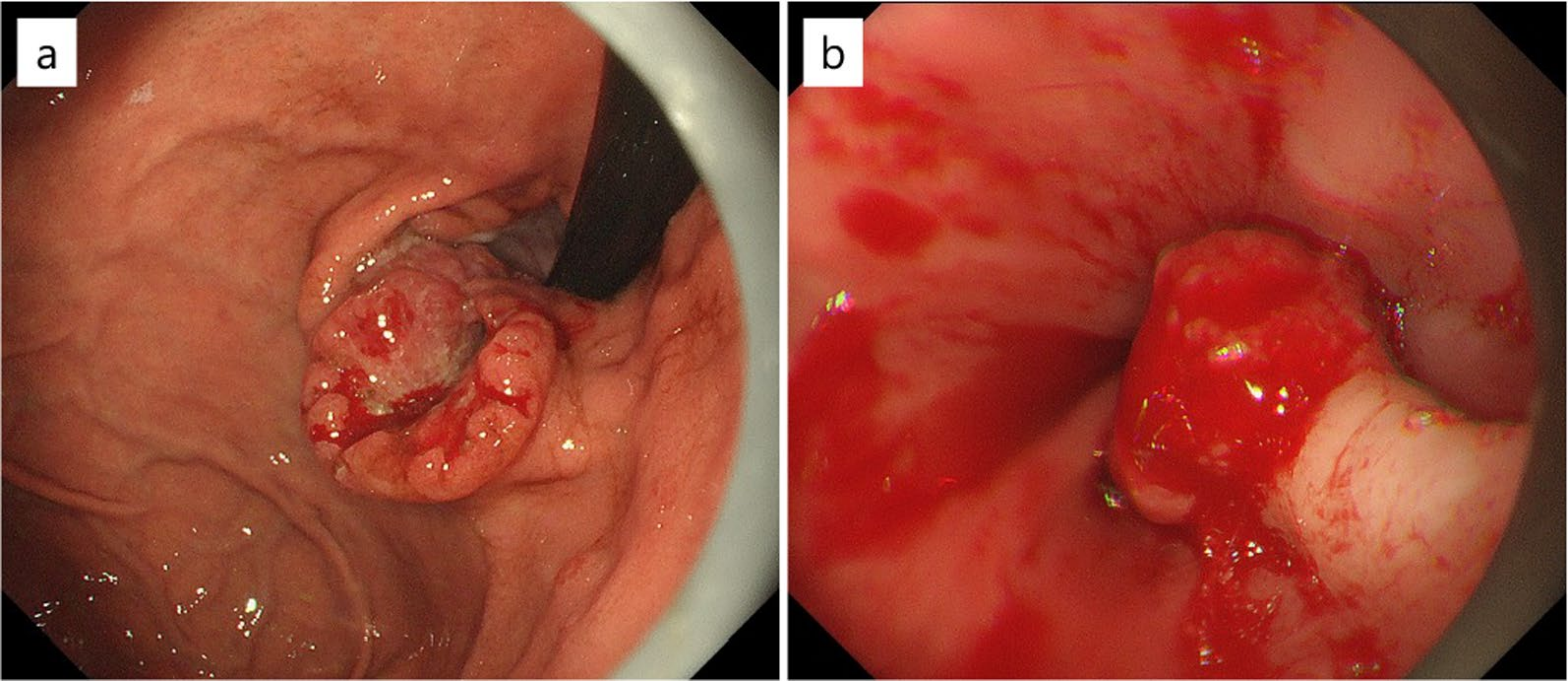
Gastroduodenal endoscopy findings. **a** A 5 cm type 2 tumor was found at the cardia side of the esophagogastric junction. **b** The tumor spread 2.5 cm from the esophagogastric junction to the esophageal side

**Fig. 2:**
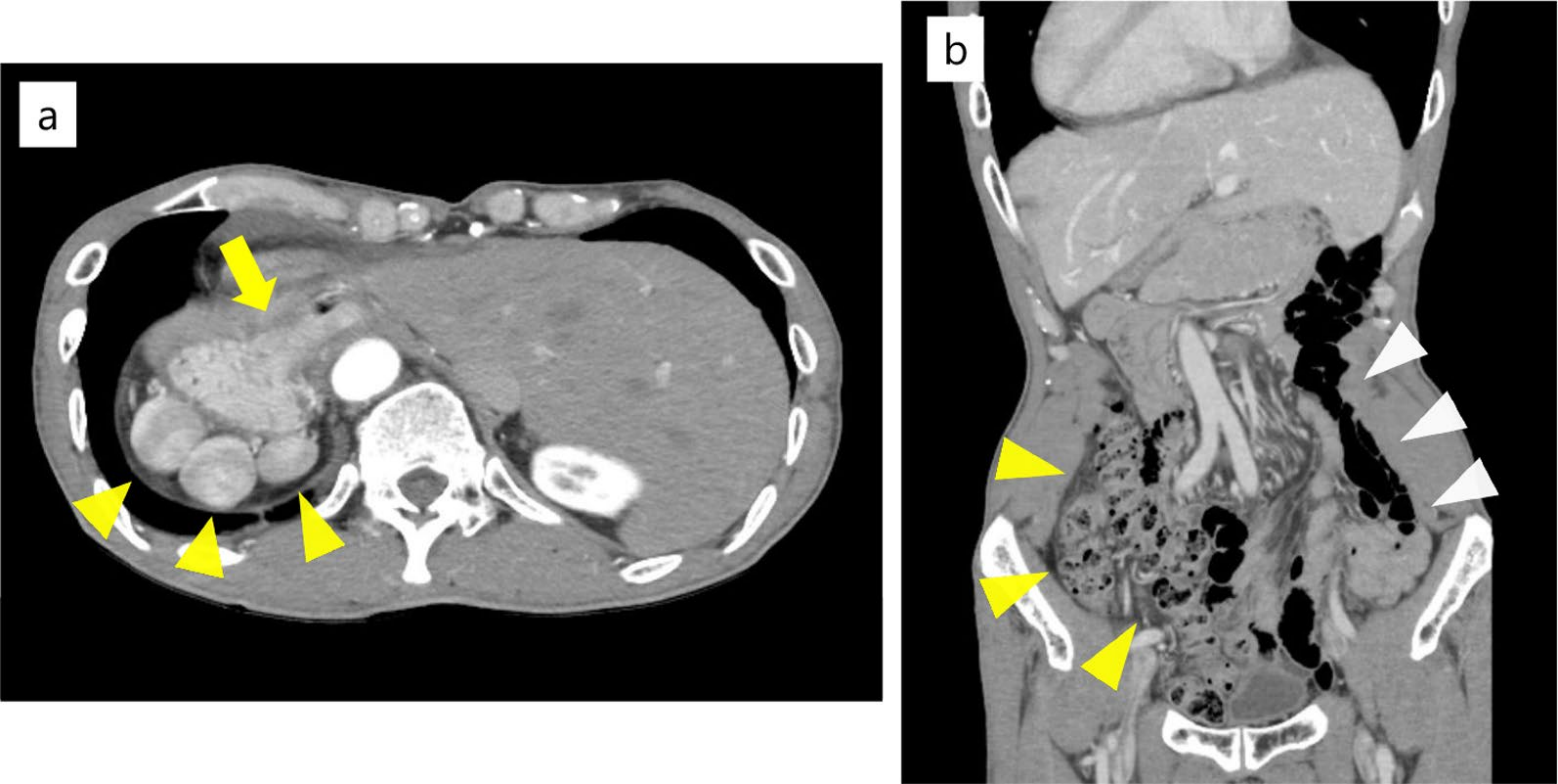
Computed tomography findings. **a** Situs inversus totalis, irregular thickening of the gastric wall (yellow arrow), and multiple spleens (yellow arrowheads) were observed. **b** The large bowel was located predominantly in the right abdomen (yellow arrowheads), and the small bowel was located predominantly in the left abdomen (white arrowheads)

**Fig. 3:**
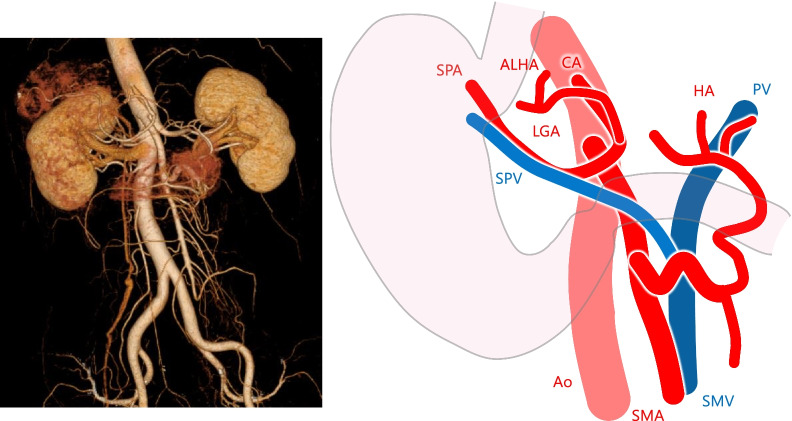
Three-dimensional reconstruction of a computed tomography angiogram revealed the absence of the common hepatic artery. In addition, the proper HA was derived from the SMA through the gastroduodenal artery, and an ALHA arose from the LGA. *SPA* splenic artery, *ALHA* accessory left hepatic artery, *CA* celiac artery, *LGA* left gastric artery, *SPV* splenic vein, *HA* hepatic artery, *PV* portal vein, *Ao* aorta, *SMA* superior mesenteric artery, *SMV* superior mesenteric vein

A 62-year-old man with SIT, intestinal malrotation, and type 2 diabetes underwent gastroduodenal endoscopy for investigation of epigastric discomfort. A 5-cm type 2 tumor was found at the cardia side of the EGJ (Fig. 1). A biopsy confirmed moderately differentiated adenocarcinoma, and the patient was diagnosed with Siewert type II EGJ cancer with 2.5 cm of esophageal involvement. Computed tomography (CT) revealed SIT, intestinal malrotation, multiple spleens, and irregular thickening of the gastric wall. No swollen lymph nodes (LNs) or distant metastases were observed (Fig. 2). The patient was diagnosed with EGJ cancer (T3N0M0 Stage IIA according to the 8th edition of the Union for International Cancer Control (UICC)-TNM classification). In addition, three-dimensional (3D) reconstruction of a CT angiogram showed that the common hepatic artery was absent, the proper hepatic artery was derived from the superior mesenteric artery through the gastroduodenal artery, and an accessory left hepatic artery (ALHA) arose from the left gastric artery (LGA) (Fig. 3). We planned a robot-assisted transhiatal lower esophagectomy and proximal gastrectomy with D2 LN dissection, including lower mediastinal lymphadenectomy.

**Fig. 4:**
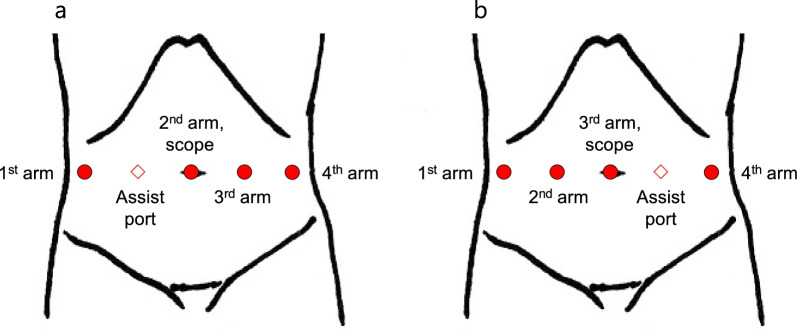
Placement of the ports and robotic arms. **a** Our routine settings. **b** This case

The patient was placed in a spinal position and the port placement mirrored our conventional settings (Fig. 4). The patient’s position was changed in a reverse Trendelenburg position with 15 degrees before the da Vinci Xi Surgical System (Intuitive Surgical, Inc., Sunnyvale, CA, USA) rolled in. The first and second arms were placed on the right side of the abdomen for Cadiere forceps and Maryland bipolar forceps, respectively. The fourth arm was placed on the left side of the abdomen for fenestrated bipolar forceps. The assistant port was also placed on the left side of the abdomen. Robotic bipolar vessel-sealing tools were attached to the second arm or fourth arm depending on the surgical site.

**Fig. 5:**
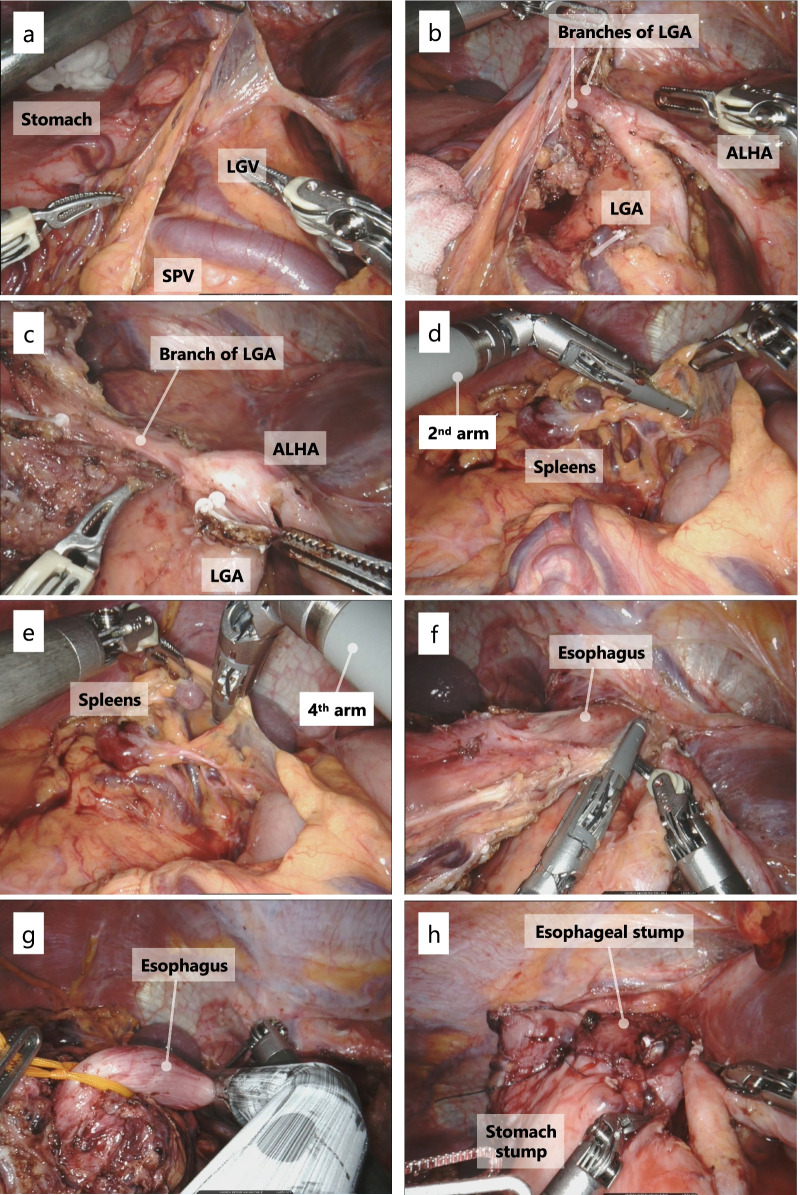
Intraoperative findings. **a** Two LGVs drained into the SPV. **b**, **c** The LGA branched an ALHA and was itself divided into three branches. **d**, **e** A sealing device was attached to the second arm and fourth arm depending on the situation. **f** Transhiatal lower mediastinal lymphadenectomy was performed. **g** The esophagus was transected 4 cm above the esophagogastric junction. **h** Esophagogastrostomy was performed using the side overlap with fundoplication by Yamashita method. *LGV* left gastric vein, *SPV* splenic vein, *LGA* left gastric artery, *ALHA* accessory left hepatic artery

After laparoscopic inspections, the lesser omentum was opened and suprapancreatic LN dissection was started. The two left gastric veins draining into the splenic vein (SPV) were clipped and cut (Fig. 5a). The LGA branched an ALHA and was itself divided into three branches. The branches of the LGA were clipped and cut, preserving the root itself (Fig. 5b, c). Station 11p and 11d LNs were dissected, tracing the splenic artery behind the SPV. Next, the greater omentum was dissected from the middle part toward the lower pole of the spleen, and station 4sa LNs were dissected. The rest of the suprapancreatic LN dissection was then completed toward the crus of the diaphragm. On the right side of the patient, the left gastroepiploic vessels and the short gastric vessels were divided by a sealing device attached to the second arm (Fig. 5d) or fourth arm (Fig. 5e) depending on the working angle. Transhiatal lower mediastinal lymphadenectomy was then performed (station 110 LNs) (Fig. 5f). We decided to secure a safety margin of at least 2 cm from the tumor. It was 4 cm from the angle of His based on preoperative esophagogastric fluoroscopy, where was transected with an EndoWrist Stapler (Intuitive Surgical, Inc., Sunnyvale, CA, USA) (Fig. 5g). The stomach was transected at the upper one-third level. The resected specimen was extracted through an umbilical incision.

After checking the margin of softy on the back table, esophagogastrostomy was performed according to the side overlap with fundoplication by Yamashita (SOFY) method as follows [7]. The central apex and left edges of the remnant stomach stump were fixated by suture to the crus of the diaphragm. The esophagus was pulled caudally, and the most proximal dorsal side of the esophagus was fixated by suture to the apex of the remnant stomach stump to prevent the esophagus from being pulled into the mediastinum. Small incisions for a stapler were made in the center of the anterior gastric wall and left side of the esophageal stump, respectively. A 45-mm EndoWrist Stapler was inserted into both holes. The esophagus was then rotated 45 degrees clockwise and stapled to suture the left wall of the esophagus to the stomach. The entry hole was closed using 3–0 absorbable barbed sutures. The esophagus was rotated back 45 degrees, and the posterior wall was placed parallel to the stomach wall. The right side of the esophagus was fixated by suture, completing the valvuloplasty (Fig. 5h).

**Fig. 6:**
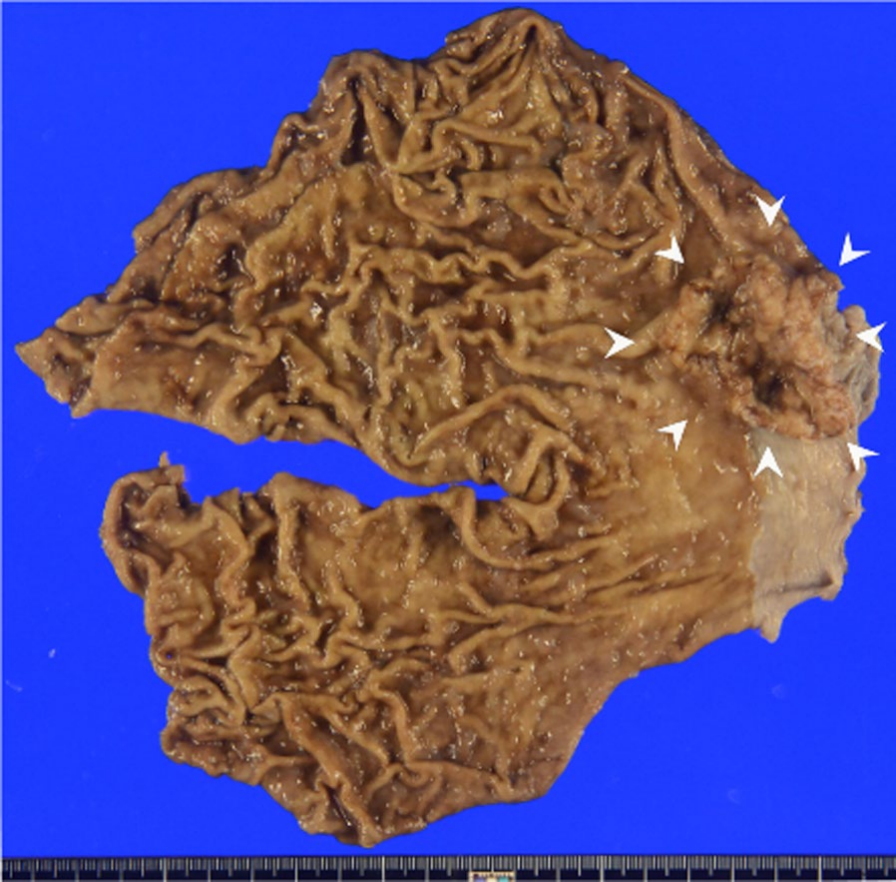
The resected specimen revealed a Siewert type II tumor measuring 50 × 37 mm in diameter (arrowheads)

The surgical time was 296 min, and the amount of blood loss was small. Histopathological diagnosis revealed a Siewert type II tumor measuring 50 × 37 mm in diameter and moderately differentiated adenocarcinoma with subserosal invasion (Fig. 6). Three metastatic LNs were present around the cardia. The final stage was pT3N2 pStage IIIB according to the 8th edition of the UICC-TNM classification. The patient had an uneventful postoperative course and was discharged 11 days after surgery.

## Discussion

This report has described a case of robot-assisted transhiatal lower esophagectomy and proximal gastrectomy for treatment of Siewert type II advanced EGJ cancer with SIT. To collect case reports on robot-assisted gastrectomy for patients with SIT, we searched PubMed for articles published from 2012 to August 2021 using the search terms “situs inversus totalis”, “robot”, “gastric cancer”, “esophagogastric junctional cancer”, and “Siewert type II cancer.” Furthermore, we read reference articles and related PubMed articles. Nine cases, including our case, were available for analysis (Table 1) [8–15]. To the best of our knowledge, this is the first report of robot-assisted transhiatal lower esophagectomy and proximal gastrectomy with esophagogastrostomy for advanced EGJ cancer with SIT.

**Table 1:** Reported cases of robotic-assisted gastrostomy for gastric cancer or esophagogastric junction cancer in patients with SIT

References	Year	Age/sex	Type of gastrectomy	Reconstruction	Lymph node dissection	Port placement	Anomalies	Vessel anomalies	Operation time (min)	Complications
[8]	2012	47 M	Distal	Billroth II	D1 +	NA	None	None	300	None
[9]	2017	52 F	Distal	Billroth I	D1 +	Mirror image	None	None	195	None
[10]	2017	60 M	Total	Roux-en-Y	D2	NA	Multiple spleens, Intestinal malrotation	Lack of CHA, RHA from SMA, ALHA from LGA	NA	None
[11]	2018	53 M	Distal	Billroth II	D2	NA	None	None	180	None
[12]	2019	80 F	Distal	Billroth I	D2	Routine positions	None	None	260	None
[13]	2020	84 M	Total	Roux-en-Y	D2	Mirror image	None	None	NA	None
[14]	2021	69 M	Distal	Roux-en-Y	D2	Routine positions	None	None	205	None
[15]	2021	71 F	Proximal	Esophagogastrostomy (double-flap technique)	D1 +	Adjusted positions	None	None	448	None
Our case	2021	62 M	Proximal (+ lower esophagectomy)	Esophagogastrostomy (SOFY)	D2	Mirror image	Multiple spleens, Intestinal malrotation	Lack of CHA, PHA from SMA, ALHA from LGA, two branches of LGV, three branches of LGA	296	None

*M* male, *F* female, *NA* not available, *CHA* common hepatic artery, *RHA* right hepatic artery, *PHA* proper hepatic artery, *SMA* superior mesenteric artery, *ALHA* accessory left hepatic artery, *LGA* left gastric artery, *LGV* left gastric vein, *SOFY* side overlap with fundoplication by Yamashita

This case provides two important clinical suggestions. The first is that we must pay special attention to vascular abnormalities during surgical treatment of patients with SIT. The second is that robotic surgery is extremely efficient even for difficult procedures in patients who have advanced EGJ cancer with SIT.

With respect to the first suggestion regarding the need for special attention to vascular abnormalities during surgery in patients with SIT, cardiovascular anomalies are reportedly 10 times more frequent in patients with SIT than in patients with normal anatomy [16]. Therefore, preoperative 3D CT angiography is important to identify any abnormal vascularization [3, 15]. In our case, preoperative 3D CT angiography showed absence of the CHA, the PHA arising from the superior mesenteric artery bridged by the gastroduodenal artery, and an ALHA branching from the LGA. However, minor abnormalities, such as three branches of the LGA and two left gastric veins draining into the SPV, could not be recognized preoperatively. Nevertheless, they were visualized by enhanced magnification and a 3D optical system, and they were then safely divided with the use of motion scaling and improved dexterity with tremor filtration. Surgeons should be aware that patients with SIT may have unidentified vessel malformations; such knowledge will help to prevent unanticipated vessel injury.

Concerning the second suggestion regarding the efficiency of robotic surgery, the following three points have been reported as advantages over conventional laparoscopic gastrectomy: (1) it is not necessary for the surgeon to change standing positions [13, 15]. (2) The surgeon can use the devices with the nondominant hand with almost the same accuracy as the dominant hand [13]. This is based on the concept of “cross-dominance”. In the present case, although the surgeon was right-handed, surgical energy devices and clip appliers were mainly used by the left hand with safety and accuracy. (3) Robotic surgery, a solo surgery, can reduce technical difficulties [12]. In this case, lower mediastinal lymphadenectomy and reconstruction were safely completed; however, these SIT procedures need to be performed in a deep, narrow, and mirror-image surgical field.

Notably, as shown in Table 1, there is no consensus regarding port placement in robot-assisted gastrectomy for patients with SIT. We placed the ports as a mirror image to our conventional settings, and the operation was successfully completed. Takeno et al. [15] described a patient who underwent robot-assisted proximal gastrectomy with the port placements adjusted to allow an approach to the esophagus and cardia located in the right upper area. Further reports should be accumulated to determine the optimal port arrangement according to associated malformations or the type of gastrectomy.

## Conclusions

We have herein reported the first case of robot-assisted transhiatal lower esophagectomy and proximal gastrectomy with esophagogastrostomy (SOFY method) for treatment of advanced EGJ cancer with SIT. We believe that robotic surgery is efficient even for complex conditions, such as Siewert type II advanced EGJ cancer with SIT.

## Data Availability

All data generated during this study are included in this published article.

## Abbreviations

SIT: Situs inversus totalis
EGJ: Esophagogastric junction
CT: Computed tomography
3D: Three-dimensional
ALHA: Accessory left hepatic artery
LGA: Left gastric artery
LN: Lymph node
SPV: Splenic vein
SOFY: Side overlap with fundoplication by Yamashita

## References

[1] González-Castillo A, Rojas S, Ortega M, Rodríguez-Baeza A (2018). Variations in vascular anatomy and unilateral adrenal agenesis in a female cadaver with situs inversus totalis. Surg Radiol Anat.

[2] Kigasawa Y, Takeuchi H, Kawakubo H, Fukuda K, Nakamura R, Takahashi T (2017). Laparoscopy-assisted distal gastrectomy in a case of gastric cancer with situs inversus totalis: a case report. Asian J Endosc Surg.

[3] Namikawa T, Maeda M, Yokota K, Tanioka N, Iwabu J, Munekage M (2021). Laparoscopic distal gastrectomy for synchronous gastric cancer and gastrointestinal stromal tumor with situs inversus totalis. In Vivo.

[4] Uyama I, Suda K, Nakauchi M, Kinoshita T, Noshiro H, Takiguchi S (2019). Clinical advantages of robotic gastrectomy for clinical stage I/II gastric cancer: a multi-institutional prospective single-arm study. Gastric Cancer.

[5] Suda K, Man-I M, Ishida Y, Kawamura Y, Satoh S, Uyama I (2015). Potential advantages of robotic radical gastrectomy for gastric adenocarcinoma in comparison with conventional laparoscopic approach: a single institutional retrospective comparative cohort study. Surg Endosc.

[6] Choi S, Song JH, Lee S, Cho M, Kim YM, Kim HI (2021). Trends in clinical outcomes and long-term survival after robotic gastrectomy for gastric cancer: a single high-volume center experience of consecutive 2000 patients. Gastric Cancer.

[7] Yamashita Y, Yamamoto A, Tamamori Y, Yoshii M, Nishiguchi Y (2017). Side overlap esophagogastrostomy to prevent reflux after proximal gastrectomy. Gastric Cancer.

[8] Kim HB, Lee JH, Park DJ, Lee HJ, Kim HH, Yang HK (2012). Robot-assisted distal gastrectomy for gastric cancer in a situs inversus totalis patient. J Korean Surg Soc.

[9] Alhossaini R, Hyung WJ (2017). Robotic assisted distal gastrectomy for gastric cancer in a patient with situs inversus totalis: with video. J Gastrointest Surg.

[10] Cao Y, Li J, Shen L, Wang J, Xia Z, Tao K (2017). Gastric cancer in a situs inversus totalis patient with multiple intestinal and vessel variations related to gastrectomy surgery: a case report and literature review. Medicine (Baltimore).

[11] Dai HB, Wang ZC, Feng XB, Wang G, Li WY, Hang CH (2018). Case report about a successful full robotic radical gastric cancer surgery with intracorporeal robot-sewn anastomosis in a patient with situs inversus totalis and a two-and-a-half-year follow-up study. World J Surg Oncol.

[12] Ojima T, Nakamura M, Nakamori M, Yamaue H (2019). Robotic distal gastrectomy with D2 lymphadenectomy for gastric cancer in a patient with situs inversus totalis. Surg Oncol.

[13] Yoshimoto T, Yoshikawa K, Tokunaga T, Nishi M, Takasu C, Kashihara H (2021). Robotic-assisted total gastrectomy in a patient with gastric cancer associated with situs inversus totalis: with video. Asian J Endosc Surg.

[14] Abbey E, Yang F, Qi L, Wu JJ, Tong L, Zhen Z (2021). Situs inversus totalis patients with gastric cancer: Robotic surgery the standard of treatment? A case report. Int J Surg Case Rep.

[15] Takeno A, Masuzawa T, Katsuyama S, Murakami K, Kawai K, Katsura Y (2021). Robotic-assisted proximal gastrectomy using the double-flap technique for early gastric cancer with situs inversus totalis: a case report. Surg Case Rep.

[16] Shibata K, Kawamura H, Ichikawa N, Shibuya K, Yoshida T, Ohno Y (2018). Laparoscopic total gastrectomy for advanced gastric cancer in a patient with situs inversus totalis. Asian J Endosc Surg.

